# Supplementary material for: Machine learning modeling of family wide enzyme-substrate specificity screens
Source: PLoS Comput Biol. 2022 Feb 10;18(2):e1009853. doi: 10.1371/journal.pcbi.1009853 (PMC8865696; doi:10.1371/journal.pcbi.1009853)
Supplement: Attachment — Submitted filename: PCOMPBIOL-D-21-01963-review.pdf [file pcbi.1009853.s002.pdf]

The study investigates the task of predicting enzymes specificities. The authors curated seven datasets from the literature, each containing a set of enzymes, substrates, and the corresponding measured activities. After pre-processing the data, the authors trained several models, namely KNN for the baseline, ANNs, and logistic regressions. ANNs were trained on the whole dataset (multi-task predictor), in which case the predictor takes the encodings of the protein and small molecule and predicts the binary label (active/not active). The logistic regressions and KNNs were trained on the subsets for each substrate (single-task predictor: enzyme discovery problem) or each enzyme (single-task predictor: substrate discovery problem). In this case, the predictor takes the enzyme or substrate features as input, respectively. The authors then conclude that the multi-task predictors do not show any significant increase in performance compared to single-task predictors based on areas under precision-recall curves, indicating that modern multi-task strategies do not seem to leverage information from multiple substrate/enzyme measurements effectively. Finally, the authors looked into different pooling strategies for enzyme encodings to show that structure-aware pooling might provide better performance of ML-based predictors compared to classical averaging.

I found this manuscript fascinating and timely as, from my experience, many enzymologists are now re-evaluating the datasets they have at hand to see if machine learning can reveal some patterns in their data, applicable for annotating yet untested enzymes and substrates. Moreover, with the recent progress in high-throughput enzyme characterization methods, we are bound to see more similar datasets in the literature in the nearest future. The authors also share their datasets and codes, which significantly contribute to the field. The insights into the compound-protein interactions and the limits of the current ML-based approaches to account for larger datasets with multiple substrates enzymes are novel. Thus, I recommend accepting this paper after addressing the following concerns:

1. The models. I was lost with the models the authors tested. In lines 138-141 and 145-149, they mention three ways of producing features for substrates (VAE-based, CPI, Morgan) x 2 architectures (concatenation or projection). However, in the caption to Fig 2, there are four models, which I struggle to align with the six mentioned before: concatenation with one-hot(?), concatenation with Morgan, dot product with Morgan, and a model called “FFN: ESM-1b”.

   Do I understand correctly that the last model does not take any substrate encoding but predicts the complete fixed set of activities instead? Please, could the authors use the same terminology for the Model section and all the graphs and tables so that readers can easily follow which model produces which results?

2. The protocol. In lines 166-167, the authors mention they selected hyper-parameters based on the thiolase dataset (similar optimization was done for the substrate discovery problem). While I understand the overfitting concern behind using this protocol, it seems that each dataset will require at least some hyper-parameter optimization. There is very little evidence of model transfer between different protein families in the literature so far; therefore, I find it difficult to justify the hyper-parameter export from the thiolase dataset onto other families from the biochemical point of view. This export could be the reason behind the low performance of the multi-task models. Could the authors also report the performance similar to Figs 2 and 3 on the thiolase and halogenase datasets for enzyme discovery and substrate discovery tasks, respectively? I would like to see if the conclusions about the comparison might be reversed there. Another test might be to evaluate the increase in performance on selected datasets if the hyper-parameter optimization is allowed locally, one dataset at a time. This will likely risk overfitting but might give at least some upper boundary to the theoretical best performance of multi-task predictors for the given range of architectures.

Minor comments:

1. Line 154-155. I would mention "for each dataset" explicitly here and whenever the authors mention "entire training set" because the latter can also be understood in the sense that all the datasets were pooled together and a single large model was trained, which I presume was not the case.
2. In Fig 1B, I would remove the icons of the substrate and enzyme next to the names of the columns and rows as those are confusing and unnecessary.
3. In the caption to Fig 2, the authors state: "a single model jointly predicts all enzyme-substrate activities with a shared intermediate…." I do not quite understand what kind of intermediate they mean. Could they please clarify?
4. Line 174 does not seem correct as, based on Fig 2B, the CPI model does outperform a single-task model on the Halogenase dataset. Also, when the authors mention "CPI models", do they mean all the multi-task models or only the "CPI" one? Because it seems the left part of Fig 2A also covers the models labeled as "Multi-task", and on Halogenase data, those seem to outperform the logistic regression.
5. Lines 244-247, I think the authors' findings also show that simple models often outperform more sophisticated ones, which is why the former should be recommended as baselines in any protein-engineering task. I can think of one more study pointing out a similar observation, which might be worth citing: arxiv.org/abs/2011.03443. On a similar note, it might be appropriate to cite DOI: 10.1093/nar/gkaa372 as a recent effort in mining new enzymes and arxiv.org/abs/2012.02679 as another study comparing different pooling strategies for protein embeddings.
6. In Figure 5B, when the number of pooled positions is 1, does it mean that just the embedding of one column of the MSA is used? I am surprised the performance is so close to the upper baseline in this case.
7. In line 407, the authors mention selecting either an active site bound ligand or active site residue from the literature. Which catalytic residue did they select when there are several of them? How did the authors ensure that the bound ligands were, in fact, bound at the active sites and not some other parts of the proteins?
8. Line 122: "they have"; line 292: "most".
