# Supplementary material for: Machine learning modeling of family wide enzyme-substrate specificity screens
Source: PLoS Comput Biol. 2022 Feb 10;18(2):e1009853. doi: 10.1371/journal.pcbi.1009853 (PMC8865696; doi:10.1371/journal.pcbi.1009853)
Supplement: Attachment — Submitted filename: Enz_sub_author_response.pdf [file pcbi.1009853.s003.pdf]

## Authors' Response to Reviews of

# Machine learning modeling of family wide enzyme-substrate specificity screens

Samuel Goldman, Ria Das, Kevin K. Yang, Connor W. Coley*
*PLOS Computational Biology, *E-mail: ccoley@mit.edu.*

---

**RC:** ***Reviewers' Comment***, AR: Authors' Response, ☐ Manuscript Text

We thank the editor and reviewers for their feedback and positive reception of our work. We have addressed all concerns in this point-by-point response and made the requisite changes to the manuscript. All relevant changes to the manuscript text are reproduced in this point-by-point response for ease of reference, with new manuscript additions highlighted in blue.

## Reviewer 1

**RC:** ***Goldman et al carry out a thorough evaluation of how machine learning might be used to predict the substrate specificity of enzymes for biocatalysis. They present two important and relevant questions; can substrate specificity be predicted from the known substrate scope of an enzyme, and can enzymes active against a given substrate be predicted from data on other enzymes. An interesting finding in this work is that utilising the data for multiple enzymes against multiple substrates does not outperform training on either a single substrate (with multiple enzymes), or a single enzyme (against multiple substrates). It might be expected that utilising all the data would result in better predictions, but this is shown not to be the case. This sets the scene for future work in this area. The authors also demonstrate an interesting approach for creating a representation of only the active site of an enzyme, which in some cases shows improved predictive performance.***

***I found the paper to be well written and structured in a logical way, and I enjoyed reading it. The authors make a valuable contribution to the field, and I believe the work will be of interest to the readership of PLOS Computational Biology. I have made a few comments which the authors might like to consider, but I otherwise recommend this work for publication.***

AR: We thank the reviewer for the positive support and feedback on our manuscript. We hope our responses and changes to the text have outlined below will further increase the clarity of our work and and address the comments raised.

**RC:** ***It might be helpful to briefly note in the methods section that the thresholds for binarization are presented in the supporting information.***

AR: We have made the following modification to our *Methods* subsection *Dataset preparation*:

> Activity binarizations are chosen to closely mirror the original dataset preparation with exact cutoff thresholds described in the Supporting Information.

**RC:** ***Why did the authors choose ESM-1b (other than being 'state of the art'). Is it expected that ESM-1b will perform better than other representations such as UniRep or SeqVec? Were other representations***

***investigated? Would there be any benefit to including the choice of sequence representation as a hyper-parameter in a machine learning pipeline? A brief description of why ESM-1b was chosen would be useful.***

AR: The prevailing pretrained protein representation learning methods (Seq2Vec [13], UniRep [1], TAPE [21], ESM-1b [23], etc.) seek to distill representations by learning the likelihood of protein sequences. We contend that ESM-1b is a reasonable choice for language model due to both its size, performance on unsupervised contact prediction, and finally its its ability to infer functional protein effects. As illustrated in Rives et al., ESM-1B contains a full order of magnitude more parameters to learn underlying information about proteins than Tape, SeqVec, and UniRep (Tab. R1). ESM-1b drastically outperforms other sequence based representation learning model in its ability to capture sequence-level structure properties, only showing comparatively low performance on remote homology, a task less relevant for protein function prediction within a single family of enzymes (Tab. R1). Rives et al. also demonstrate that their ESM-1b based approach is not only best in class on previously enumerated protein structural tasks, but also on protein variant effect prediction by testing their approach on mutational effect prediction against the Envision dataset [12]. For similar reasons, other parallel projects seeking to benchmark and understand the limitations of protein-based machine learning have also converged upon ESM-1b as a reasonable choice for representation learning when large sweeps over all possible embeddings are out-of-scope [8].We add the following in-text clarification:

> Pre-trained representations are well-suited to low data tasks and have been applied to protein property prediction[4, 23, 1] as well as compound protein interaction[17]. While there has been an explosion in available pre-trained protein representations including UniRep [1], SeqVec [13], MT-LSTM [2, 3], ESM-1b stands out in its scale (i.e., number of parameters and training sequences), performance on contact prediction tasks, ease of use, and also ability to effectively predict the functional effect of sequence variations [23].

Table R1: Summary of relative language modeling approaches reproduced from Rives et al [23].

| Model | Params | Remote homology | Secondary structure prediction | Contact prediction |
|---|---|---|---|---|
| UniRep [1] | 18 M | 0.527 | 18.4 | 21.9 |
| SeqVec [13] | 93 M | 0.545 | 62.1 | 29.0 |
| TAPE [21] | 38 M | 0.581 | 58.0 | 23.2 |
| ESM-1b [23]] | 652.4 M | 0.532 | 71.6 | 56.9 |

Lastly, in earlier versions of this work we also considered utilizing representations from Bepler et al. [2] that were *directly trained on structure prediction task*. We found representations from ESM-1b to be overall stronger and to avoid adding confusion over embedding choices have not included these results in our manuscript (Tab. R2).

Table R2: AUPRC results on the esterase and phosphatase datasets when predicting held out enzymes. For the two datasets with the largest number of enzymes, ESM embeddings outperform those of Bepler et al.

| | **Dataset** | Esterase | Phosphatase |
|---|---|---|---|
| **Method Type** | **Method** | | |
| **Single-task** | **Ridge: pretrained (ESM)** | $\mathbf{0.584 \pm 0.001}$ | $\mathbf{0.414 \pm 0.001}$ |
| | **Ridge: pretrained (Bepler)** | $0.490 \pm 0.001$ | $0.329 \pm 0.001$ |

**RC:** ***For the CPI models, what is the rational for taking the dot product of the substrate and sequence representations for predictions? Also, how were the representations projected into smaller and equal length vectors (line 148)?***

AR: To effectively generalize to either new compounds or new proteins in a CPI model, the architecture must jointly utilize the two separate embeddings. While concatenating the two representations is a natural choice and utilized in certain CPI-based architectures [14], other prior works take the dot product between featurizations for both a protein and compound representation [18]. Dot product based approaches require no additional parameters, a desirable feature for lower-N data prone to over-fitting. On the other hand, concatenation requires an additional top-layer or multi layer perception model upon the concatenation. Thus, we tested both architecture choices and optimized hyperparameters for each architecture independently.

We have updated the text to explain how the features are first projected into equal length vectors:

> To attempt to model interactions, we consider two simple top models inspired by the CPI literature[14, 18, 17] that either (1) concatenate the representations of the enzyme and substrate before applying a shallow feed forward neural network or (2) project the representations of the enzyme and substrate to smaller and equal length vectors using a shallow multi-layer perceptron (MLP) before taking their dot product (Fig. 1D).

**RC:** ***Why was Levenshtein distance used for the KNN studies? Could a standard percentage sequence identity as is commonly reported in BLAST searches be used instead, and might this perform better given it takes into account similarities between different amino acids via a BLOSUM scoring matrix? For sequence similarity networks often an 'alignment score' is used, which may also perform better? (https://doi.org/10.1016/j.bbapap.2015.04.015)***

AR: We selected the Levenshtein distance (unweighted global alignment score) for our KNN baseline to follow precedents set by recent studies for machine learning guided AAV design [5] and enzyme engineering [4]. In addition, BLAST scores scores utilize local, not global alignments, and prioritize heuristics to increase speed.

To confirm that BLAST scores would not significantly change our results, we re-tested the nearest neighbor benchmark using BLAST E-values as distances on the two largest sequence datasets. We observed that there was little statistical difference in the results, increasing our confidence in Levenshtein distance as a reasonable sequence similarity baseline method (Tab. R3).

Table R3: PSAR results demonstrating differences between Levenshtein and BLAST distance metrics

| | **Dataset** | Esterase | Phosphatase |
|---|---|---|---|
| **Method Type** | **Method** | | |
| **Baselines** | **KNN: BLAST** | $0.528 \pm 0.001$ | $0.317 \pm 0.003$ |
| | **KNN: Levenshtein** | $0.528 \pm 0.002$ | $0.316 \pm 0.004$ |
| **Single-task** | **Ridge: ESM-1b** | $\mathbf{0.583 \pm 0.003}$ | $\mathbf{0.413 \pm 0.005}$ |

We have updated the text to explain this choice:

> We observe that our supervised models using pretrained protein representations are in fact able to outperform a nearest neighbor sequence-similarity baseline ("KNN: Levenshtein") that uses the Levenshtein distance, a simple unweighted global alignment distance used in recent protein engineering studies [5, 4], to predict held out enzyme activity (Fig. 2B, Tab. S5,S6).

**RC:** ***Line 187 to 194 – should Fig 2E be referenced here?***

AR:

> Plotting the AUPRC metric as a function of number of active enzyme-substrate pairs reveals a strong positive correlation, validating that the number of hits observed in the training set will largely determine the success of the model in generalizing beyond the training set (Fig. 2E).

**RC:** ***SI Fig 7 onwards – what do the colours mean?***

AR: We thank the reviewer for pointing out this lack of scale on our plots and have updated all appendix prediction results to include a colorbar indicating the scale for predicted activity values.

**RC:** ***Line 292 – Typo in word most***
***Line 345 – cross leave one out cross validation***

AR:

> Similarly, for the esterase dataset, coverage pooling is far more effective, indicating that a combination of targeted pooling residue strategies may be most effective (Fig. S6).

> ...trials of leave-one-out cross validation on the thiolase dataset and halogenase dataset for enzyme discovery and substrate discovery tasks, respectively...

## Reviewer 2

**RC:** ***The study investigates the task of predicting enzymes specificities. The authors curated seven datasets from the literature, each containing a set of enzymes, substrates, and the corresponding measured activities. After pre-processing the data, the authors trained several models, namely KNN for the baseline,***

***ANNs, and logistic regressions. ANNs were trained on the whole dataset (multi-task predictor), in which case the predictor takes the encodings of the protein and small molecule and predicts the binary label (active/not active). The logistic regressions and KNNs were trained on the subsets for each substrate (single-task predictor: enzyme discovery problem) or each enzyme (single-task predictor: substrate discovery problem). In this case, the predictor takes the enzyme or substrate features as input, respectively. The authors then conclude that the multi-task predictors do not show any significant increase in performance compared to single-task predictors based on areas under precision-recall curves, indicating that modern multi-task strategies do not seem to leverage information from multiple substrate/enzyme measurements effectively. Finally, the authors looked into different pooling strategies for enzyme encodings to show that structure- aware pooling might provide better performance of ML-based predictors compared to classical averaging.***

***I found this manuscript fascinating and timely as, from my experience, many enzymologists are now re- evaluating the datasets they have at hand to see if machine learning can reveal some patterns in their data, applicable for annotating yet untested enzymes and substrates. Moreover, with the recent progress in high- throughput enzyme characterization methods, we are bound to see more similar datasets in the literature in the nearest future. The authors also share their datasets and codes, which significantly contribute to the field. The insights into the compound-protein interactions and the limits of the current ML-based approaches to account for larger datasets with multiple substrates enzymes are novel. Thus, I recommend accepting this paper after addressing the following concerns:***

AR: We thank the reviewer for the positive support and for the constructive feedback on our manuscript. We hope the responses outlined below and new computational results will satisfy the concerns that were raised.

**RC:** ***The models. I was lost with the models the authors tested.***

AR: We agree that our description of model classes and models was cursory and could be difficult to understand. We have added additional information to further clarify the various architectures and comparisons detailed in our responses below.

**RC:** ***In lines 138-141 and 145-149, they mention three ways of producing features for substrates (VAE-based, CPI, Morgan) x 2 architectures (concatenation or projection).***

AR: We first specify that lines 138-141 only indicate two featurization schemes, with one of the featurizations was inspired by a recent CPI approach:

> To featurize substrates, we test two primary featurizations: a pretrained Junction-Tree Variational Auto-Encoder (JT-VAE)[16] and the widely used Morgan circular fingerprints (1024 bits) [20]. Despite pre-training compound representations having only marginal benefits on property prediction tasks [15], a recent CPI study [14] extracted compound representations from a pre-trained JT-VAE model and utilized these to identify new kinase inhibitors. Due to the closeness in our proposed task, we follow their methodology and extract the same embeddings for substrates to compare against Morgan fingerprints.

Second, in lines 145-149, we add an additional description of all the other model classes against which we compare. We further reference the reader to a new section within the Supporting Information that describes each model class in more depth. A reproduction of the new supporting information table can be found in this response (Tab. R4).

> To attempt to model interactions, we consider two simple top models inspired by the CPI literature[14, 18, 17] that either (1) concatenate the representations of the enzyme and substrate before applying a shallow feed forward neural network or (2) project the representations of the enzyme and substrate to smaller and equal length vectors using a shallow multi-layer perceptron (MLP) before taking their dot product (Fig. 1D). To evaluate these CPI based architectures, we consider three other model classes for comparison: baselines utilizing simple similarity across enzymes and substrates for prediction; multi-task models that learn to predict activity for enzymes against substrates simultaneously but without any feature information about the substrates themselves (and *vice versa* for substrate discovery); and single-task models with no information sharing across substrate (enzyme) tasks. We refer the reader to the Supporting Information for a more complete description of all model classes evaluated (Tab. S3).

**RC:** ***However, in the caption to Fig 2, there are four models, which I struggle to align with the six mentioned before: concatenation with one-hot(?), concatenation with Morgan, dot product with Morgan, and a model called "FFN: ESM-1b". Do I understand correctly that the last model does not take any substrate encoding but predicts the complete fixed set of activities instead? Please, could the authors use the same terminology for the Model section and all the graphs and tables so that readers can easily follow which model produces which results?***

AR: The reviewer is correct in understanding that the last multi-task model predicts the complete, fixed-length set of activities for each enzyme against all substrates. We have updated the portion of the caption to be more consistent with the figure labels themselves:

> . . . AUPRC is compared on five different datasets, arranged from left to right in order of increasing number of enzymes in the dataset. Baseline models are compared against multi-task models, CPI models, and single-task models. K-nearest neighbor (KNN) baselines are calculated using Levenshtein edit distances to compare sequences; multi-task models use a shared feed forward network (FFN) to compute predictions against all substrate targets, CPI models utilize FFN with either concatenation ("[{prot repr.}, {sub repr.}]") or dot product interactions ("{prot repr.}•{sub repr.}"), and ridge regression is used for single-task models.

Coupled with the new section in the supporting information (reproduced in Tab. R4), we are hopeful that this will clarify the exact descriptions of models utilized.

Table R4: New *Supporting Information* summary and description of models.

| Model class | Model | Enz. features | Sub. features | Enz. discovery? | Sub. discovery? | Model name |
|---|---|---|---|---|---|---|
| Baseline | KNN | Sequence | - | Yes | No | KNN: Levenshtein |
| | KNN | - | Morgan | No | Yes | KNN: Tanimoto |
| | Ridge | - | Random | No | Yes | Ridge: random feats. |
| Multi-task | FFN | ESM-1b | - | Yes | No | FFN: ESM-1b |
| | FFN (concat.) | ESM-1b | One-hot | Yes | No | FFN: [ESM-1b, one-hot] |
| | FFN | - | Morgan | No | Yes | FFN: Morgan |
| | FFN (concat) | One-hot | Morgan | No | Yes | FFN: [one-hot, Morgan] |
| CPI | FFN (concat) | ESM-1b | Morgan | Yes | Yes | FFN: [ESM-1b, Morgan] |
| | FFN (dot) | ESM-1b | Morgan | Yes | yes | FFN: ESM-1b • Morgan |
| Single-task | Ridge | ESM-1b | - | Yes | No | Ridge: ESM-1b |
| | Ridge | - | Morgan | No | Yes | Ridge: Morgan |
| | Ridge | - | JT-VAE | No | Yes | Ridge: JT-VAE |

**RC:** ***The protocol. In lines 166-167, the authors mention they selected hyper-parameters based on the thiolase dataset (similar optimization was done for the substrate discovery problem). While I understand the overfitting concern behind using this protocol, it seems that each dataset will require at least some hyper-parameter optimization. There is very little evidence of model transfer between different protein families in the literature so far; therefore, I find it difficult to justify the hyper- parameter export from the thiolase dataset onto other families from the biochemical point of view.***

AR: We agree that there is little biochemical justification to sharing parameters across different biochemical families and tasks. However, we believe exporting simple hyperparameters across the tasks is a reasonable step, given the limited set of hyperparameters we test. We note that hyperparameters only control a small number of model attributes:

1. KNN models:
   (a) Number of nearest neighbors
2. FFN models:
   (a) Hidden dimension
   (b) Layers
   (c) Dropout
   (d) Learning rate
   (e) Weight decay
3. Ridge models:
   (a) Regularization coefficient

Previous authors have made similar assumptions around hyperparameters across protein families. when conducting mutation effect prediction, Rives et al. do not change hyperparameters across their 42 deep mutational scan dataset [23]. As the reviewer notes, such a decision can help safeguard against overfitting, eliminate additional noise in hyperparameter searching, and prevent the scope of such a study across datasets from becoming prohibitively computationally expensive.

**RC:** ***This export could be the reason behind the low performance of the multi-task models. Could the authors also report the performance similar to Figs 2 and 3 on the thiolase and halogenase datasets for enzyme discovery and substrate discovery tasks, respectively? I would like to see if the conclusions about the comparison might be reversed there.***

AR: We have re-run the full evaluation on the thiolase and halogenase datasets for enzyme and substrate discovery respectively. To include this new information, we have updated the Supporting Information results tables to now feature an additional column for the hyperparameter optimization dataset (Tab. R5, R6) and have updated the main text to reference these tables when describing hyperparameter optimization.

> We optimize model hyperparameters on the thiolase dataset[24] prior to training and evaluating on the remaining five datasets. We additionally report benchmarking performance for the thiolase dataset in the Supporting Information (Tab. S5, S6).

> In this case, we restrict our analysis only to the glycosyltransferase and phosphatase datasets where the number of substrates is $> 50$, using the halogenase dataset to tune hyperparameters for each model. We report the results comparison on the halogenase dataset in the Supporting Information (S7, S8).

In the enzyme discovery direction, we find that multi-task models and CPI models are the top performing models on the thiolase dataset (used for hyperparameter optimization), similar to halogenases. However, we suspect that this is likely a feature of the dataset, not the hyperparameter protocol, as the halogenase dataset shows a similar trends in enzyme discovery. Multi-task models with no information of the substrate are tied with CPI-based models for thiolases. This indicates that the CPI based models are still not able to learn more complex interactions as a function of the substrate structure.

When considering the halogenase data (dataset used for hyperparameter optimization) in the substrate discovery setting, we find the single-task models to be most performant, consistent with results across other substrate discovery datasets.

To further validate that our observations are not an artifact of our hyperparameter export, we carry out the reviewer's second suggested test below to demonstrate how hyperparameter optimizing on a different dataset does not change our conclusions.

Table R5: Full enzyme discovery area under the precision recall curve (AUPRC) results reproduced from Supporting Information and extended with thiolase daata

| Method Type | Dataset<br>Method | BKACE | Esterase | Glyco. | Halogenase | Phosphatase | Thiolase |
|---|---|---|---|---|---|---|---|
| **Baselines** | **KNN: Levenshtein** | $0.564 \pm 0.011$ | $0.528 \pm 0.002$ | $0.539 \pm 0.003$ | $0.375 \pm 0.014$ | $0.316 \pm 0.004$ | $0.499 \pm 0.001$ |
| **CPI** | **FFN: [ESM-1b, Morgan]** | $0.478 \pm 0.012$ | $\mathbf{0.579 \pm 0.007}$ | $\mathbf{0.581 \pm 0.008}$ | $\mathbf{0.489 \pm 0.025}$ | $0.386 \pm 0.006$ | $0.536 \pm 0.027$ |
| | **FFN: ESM-1b • Morgan** | $0.645 \pm 0.007$ | $\mathbf{0.588 \pm 0.011}$ | $0.559 \pm 0.010$ | $0.463 \pm 0.021$ | $0.389 \pm 0.002$ | $\mathbf{0.541 \pm 0.003}$ |
| **Multi-task** | **FFN: [ESM-1b, one-hot]** | $0.433 \pm 0.007$ | $0.557 \pm 0.007$ | $0.526 \pm 0.024$ | $\mathbf{0.510 \pm 0.043}$ | $0.361 \pm 0.008$ | $\mathbf{0.552 \pm 0.013}$ |
| | **FFN: ESM-1b** | $\mathbf{0.664 \pm 0.011}$ | $0.572 \pm 0.008$ | $0.543 \pm 0.024$ | $0.420 \pm 0.003$ | $0.359 \pm 0.005$ | $0.487 \pm 0.003$ |
| **Single-task** | **Ridge: ESM-1b** | $0.648 \pm 0.011$ | $\mathbf{0.583 \pm 0.003}$ | $\mathbf{0.575 \pm 0.000}$ | $0.446 \pm 0.000$ | $\mathbf{0.413 \pm 0.005}$ | $0.519 \pm 0.000$ |

Table R6: Full substrate discovery area under the precision recall curve (AUPRC) results reproduced from the Supporting Information now includes halogenase results used for hyperparameter optimization

| Method Type | Dataset<br>Method | Esterase | Glyco. | Halogenase | Phosphatase |
|---|---|---|---|---|---|
| **Baselines** | **KNN: Tanimoto** | $0.609 \pm 0.008$ | $0.588 \pm 0.006$ | $0.464 \pm 0.000$ | $0.426 \pm 0.002$ |
| | **Ridge: random feats.** | $0.327 \pm 0.014$ | $0.239 \pm 0.032$ | $0.258 \pm 0.037$ | $0.294 \pm 0.006$ |
| **CPI** | **FFN: [ESM-1b, Morgan]** | $0.674 \pm 0.019$ | $0.673 \pm 0.010$ | $0.471 \pm 0.005$ | $0.462 \pm 0.006$ |
| | **FFN: ESM-1b • Morgan** | $\mathbf{0.709 \pm 0.018}$ | $\mathbf{0.693 \pm 0.005}$ | $0.478 \pm 0.033$ | $\mathbf{0.506 \pm 0.004}$ |
| **Multi-task** | **FFN: Morgan** | $0.654 \pm 0.011$ | $0.689 \pm 0.005$ | $0.362 \pm 0.019$ | $0.442 \pm 0.001$ |
| | **FFN: [one-hot, Morgan]** | $\mathbf{0.707 \pm 0.019}$ | $0.677 \pm 0.009$ | $0.469 \pm 0.009$ | $0.493 \pm 0.005$ |
| **Single-task** | **Ridge: Morgan** | $\mathbf{0.716 \pm 0.010}$ | $\mathbf{0.699 \pm 0.008}$ | $\mathbf{0.525 \pm 0.000}$ | $\mathbf{0.504 \pm 0.002}$ |
| | **Ridge: JT-VAE** | $0.489 \pm 0.004$ | $0.505 \pm 0.007$ | $0.468 \pm 0.000$ | $0.411 \pm 0.002$ |

**RC:** ***Another test might be to evaluate the increase in performance on selected datasets if the hyper-parameter optimization is allowed locally, one dataset at a time. This will likely risk overfitting but might give at least some upper boundary to the theoretical best performance of multi-task predictors for the given range of architectures.***

AR: We perform the suggested test on our largest datasets with the least noise in model results, the phosphatase dataset. Specifically, we consider the extrapolation direction of enzyme discovery and carry out a round of hyperparameter optimization with all models tested.

First, we note that hyperparameters selected are quite similar across the thiolase and phosphatase optimization loops indicating that the datasets appear to have similar properties (Tab. R7).

Next, we compare the results themselves on the phosphatase dataset when using the hyperparameters discovered by both optimization loops. We repeat benchmarking experiments on the phosphatase dataset itself with these new parameters as suggested by the reviewer. We find that performance does not signfiicantly change. The strength of this result increases our confidence in exporting hyperparameters from a single held out dataset to other datasets of comparable size.

Table R7: Enzyme discovery hyperparameter comparison. Hyperparameters discovered by the Optuna framework on both the thiolase and phosphatase datasets are shown.

| Model | Hyperparameter | Thiolase hyperparameter | Phosphatase hyperparameter |
|---|---|---|---|
| **KNN** | **Neighbors** | 7 | 5 |
| **Ridge** | **Log alpha** | 2 | 2 |
| **FFN (multi-task)** | **Dropout** | 0.15 | 0.15 |
| | **Weight decay** | 0.0006 | 0.001 |
| | **Hidden dimension** | 50 | 90 |
| | **Hidden layers** | 1 | 1 |
| | **Learning rate** | 0.0001 | 0.001 |
| **FFN (concat)** | **Dropout** | 0.138 | 0.05 |
| | **Weight decay** | 0.007 | 0.005 |
| | **Hidden dimension** | 30 | 50 |
| | **Hidden layers** | 1 | 1 |
| | **Learning rate** | 0.0001 | 0.0002 |
| **FFN (dot)** | **Dropout** | 0.04 | 0.2 |
| | **Weight decay** | 0.001 | 0.001 |
| | **Hidden dimension** | 90 | 90 |
| | **Hidden layers** | 2 | 1 |
| | **Learning rate** | 0.0002 | 0.0004 |

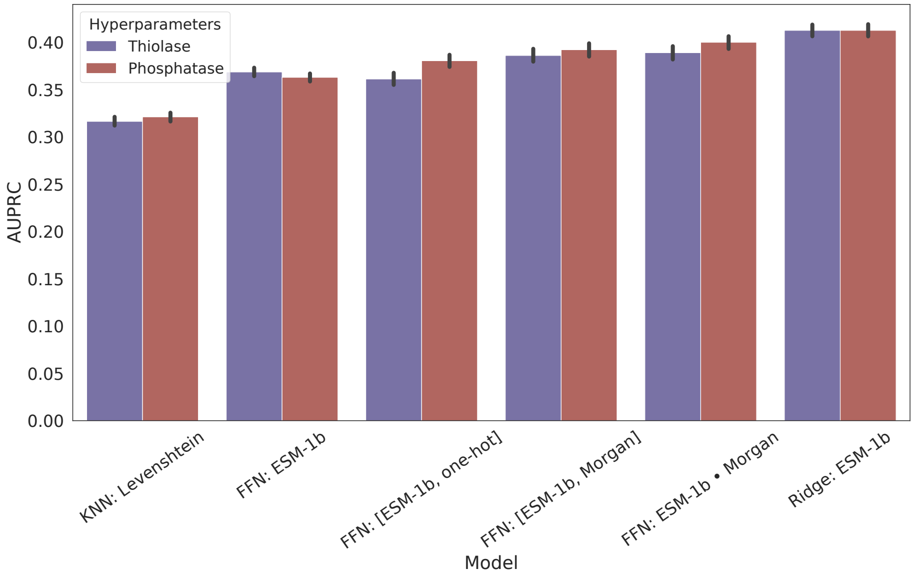


Figure R1: Enzyme discovery hyperparameter comparison on phosphatase data. Phosphatase enzyme discovery experiments are re-run exactly in the main text, using either hyperparameters found by optimization on the thiolase data (purple) or hyperparameters discovered on this data itself (red).

**RC:** ***Line 154-155. I would mention "for each dataset" explicitly here and whenever the authors mention "entire training set" because the latter can also be understood in the sense that all the datasets were pooled together and a single large model was trained, which I presume was not the case.***

AR: We agree that this clarification will make clearer that separate models are trained on each dataset and have revised the text accordingly.

> For each dataset, we train the CPI model architectures described above jointly on the entire training set.

**RC:** ***In Fig 1B, I would remove the icons of the substrate and enzyme next to the names of the columns and rows as those are confusing and unnecessary.***

AR: We have updated the figure to now feature a simplified schematic in the main text.

**RC:** ***In the caption to Fig 2, the authors state: "a single model jointly predicts all enzyme-substrate activities with a shared intermediate...." I do not quite understand what kind of intermediate they mean. Could they please clarify?***

AR: We have amended the text introducing this concept in the models section and the caption respectively:

> To attempt to model interactions, we consider two simple top models inspired by the CPI literature[14, 18, 17] that either (1) concatenate the representations of the enzyme and substrate before applying a shallow feed forward neural network or (2) project the representations of the enzyme and substrate to

> smaller and equal length vectors using a shallow multi-layer perceptron (MLP) before taking their dot product (Fig. 1D).

> ... multi-task models use a shared feed forward network (FFN) to compute predictions against all substrate targets ...

**RC:** ***Line 174 does not seem correct as, based on Fig 2B, the CPI model does outperform a single-task model on the Halogenase dataset. Also, when the authors mention "CPI models", do they mean all the multi-task models or only the "CPI" one? Because it seems the left part of Fig 2A also covers the models labeled as "Multi-task", and on Halogenase data, those seem to outperform the logistic regression.***

AR: We agree with the reviewer that we should be more careful and clear to discuss the multi-task performance results and have updated the text to reflect such a change:

> Surprisingly, however, CPI models do not outperform single-task models trained with simple logistic regression ("Ridge: ESM-1b") (Fig. 2B). Multi-task models offer a slight benefit on the halogenase dataset, but fail to outperform single-task models across the other four enzyme families tested. Further, upon closer inspection, the comparative performance of CPI based models on the halogenase dataset seem to be driven by relative performance increases only on a small number of substrate tasks as demonstrated by the upper outliers in Fig. 2C. This is despite the CPI (and multi-task) models...

**RC:** ***Lines 244-247, I think the authors' findings also show that simple models often outperform more sophisticated ones, which is why the former should be recommended as baselines in any protein- engineering task. I can think of one more study pointing out a similar observation, which might be worth citing: arxiv.org/abs/2011.03443.***

AR: We have added this conclusion more explicitly and included a citation to Shanehsazzedeh et al.:

> Our findings are retrospective... truly learn interactions. This further reinforces the necessity for simple baseline models in protein engineering studies [25, 19].

**RC:** ***On a similar note, it might be appropriate to cite DOI: 10.1093/nar/gkaa372 as a recent effort in mining new enzymes and arxiv.org/abs/2012.02679 as another study comparing different pooling strategies for protein embeddings.***

AR: We thank the reviewer for calling attention to these important recent works and have added additional citations to both in the relevant discussion around enzyme mining and improving enzyme discovery models respectively.

**RC:** ***In Figure 5B, when the number of pooled positions is 1, does it mean that just the embedding of one column of the MSA is used? I am surprised the performance is so close to the upper baseline in this case.***

AR: This is a correct interpretation of our method. While it's true that the embedding is only sampled at a small number of positions at the left-most portion of the graphs in Fig. 5B, because the positional representation is computed as a function of the entire sequence, we find it highly plausible that that the embedding at each position is representative of both neighboring amino acid embeddings and the pooled representation of the entire sequence.

**RC:** ***In line 407, the authors mention selecting either an active site bound ligand or active site residue from the literature. Which catalytic residue did they select when there are several of them? How did the authors ensure that the bound ligands were, in fact, bound at the active sites and not some other parts of the proteins?***

AR: We have updated the main text to indicate that we at times selected *multiple* active site residues to account for such instances. We refer the reviewer to Table S1 indicating that in the esterase enzyme family, we selected residues S105, D187, and H224 from structure PDB:5A6V and compute active site shells radiating out from each active site residue jointly.

We referenced the primary literature to identify such structures and manually verified that the small radius of residues around the identified active site was conserved within the multiple sequence alignment. However, this is not exhaustive and we find it likely that our strategy does not select the optimal set of informative residues for pooling, representing one possible avenue for future work.

**RC:** ***Line 122: "they have"***
***Line 292: "most".***

AR: We have updated the text accordingly for both inline edits and thank the reviewer for calling attention to these typos.

## Reviewer 3

**RC:** ***In this work, the authors use protein language model embeddings, along with substrate encodings to predict compound protein interactions. They work with multiple top models which incorporate multiple chemical encoding methods and concatenation approaches and tested with different dataset. The use of protein language model, chemical encodings and different machine learning models shows an innovative integration of many works in the literature. The proposed structure-based strategy for pooling residue representations improves CPI predictions, which has many applications in biocatalysis. This work is very well written and will form an extremely important contribution to not only the ML community but also for the biocatalysis, protein engineering and functional genomics community. I should also commend, applaud and thank the authors for providing the datasets that were used in the paper. That is significant contribution though the code for some of the methods were not clear in the GitHub repository. Please see if you can add these details as well. Taken together this paper is highly valuable though it can be strengthened further based on the following comments.***

AR: We thank the reviewer for their positive feedback and review of our work. We have further improved our work by incorporating your suggestions, and we include a detailed line-by-line summary of our changes below.

In addition, we want to make clear that we included two separate Github pages with this work. The reviewer references our dataset GitHub repository (`https://github.com/samgoldman97/enzyme-datasets`). We have included a separate GitHub repository with instructions to reproduce and replicate all experiments, figures, and tables in our paper that can be accessed here: `https://github.com/samgoldman97/enz-pred`.

**RC:** ***The introduction provides a nice description of the background but it could use more information on the protein sequence modeling such as the NLP methods used for prediction. I would urge authors to cut down the background on the enzyme side and add some paragraphs describing the protein sequence modeling (the different language models used and their success in enabling protein property prediction)***

***which forms the foundation for their use in this specific sequence to function task. Similarly, there is some background on the substrate representations that could be relevant. This could also be a place to rationalize their choice of language model (ESM1b) and the substrate representations (JT-VAE).***

AR: We have expanded our treatment of protein language modeling in the introduction:

> MLDE demonstrations often follow a similar paradigm: given a screen of a single enzyme with mutations at select positions, predict the function or activity of enzymes with new mutations. Further developments in pretrained machine learning models can now provide meaningful embeddings at single amino acid positions that capture contextual and structural information about the protein from sequence alone [2, 3, 21, 22, 23, 1]. Pre-trained machine learning models of protein sequences, specifically masked language models adopted from natural language processing [11] are trained to predict the identity of "masked" input tokens (i.e. amino acids). In doing so, the model learns a meaningful intermediate representation of the protein and distill important structural context around each amino acid position. This intermediate layer can then be extracted and treated as a fixed embedding of the protein [9]. These pre-trained embeddings have proved especially useful for the application of MLDE...

In addition, to justify the use of ESM-1b as an embeddings choice, we have added the following explanation to the text in discussing our choice of featurizations:

> Pre-trained representations are well-suited to low data tasks and have been applied to protein property prediction[4, 23, 1] as well as compound protein interaction[17]. While there has been an explosion in available pre-trained protein representations including UniRep [1], SeqVec [13], MT-LSTM [2, 3], ESM-1b stands out in its performance on contact prediction tasks, ease of use, and also ability to effectively predict the functional effect of sequence variations, likely enabled by its comparatively large scale (i.e., number of parameters and training sequences) [23].

We select the JT-VAE embeddings specifically because they were utilized by Hie et al., whose work suggested that such pre-trained embeddings could be used to improve CPI-based performance.

**RC:** ***The dataset published on GitHub is clear and well-organized. And results of CPI as well as structure-based classification models are clearly shown. However, results of regression models are not fully shown. For example, in figure 5B, Spearman's R is only plotted for Kinase dataset, while phosphatase dataset also contains activities (numeric values) for regression models. The Spearman's R's for predicting CPI of phosphatases and halogenases activities are not shown or discussed.***

AR: While many of these datasets can be treated as *either* regression or classification datasets, we make the strategic decision to model all datasets we aggregate from the literature as binary classification:

> Enzymatic catalysis (e.g., yield, conversion, activity) in each study was measured using some combination of coupled assay reporters, mass spectrometry, or fluorescent substrate readouts. Data was binarized such that every measured pair is either labeled as active (1) or inactive (0) at thresholds according to standards described in the original papers (Methods).

We choose this framing in large part because our primary application of interest was the discovery of "hook" enzymes for a new substrate, requiring only moderate activity on a substrate of interest. Further, in several of the studies, such as for the phosphatase dataset, technical replicates feature substantial noise, indicating that regression may not be most effective, which we hope to smooth by binarizing the data.

Consequently, we report AUPRC and AUC-ROC (Supporting Information) for classification datasets. We only consider Spearman's $\rho$ for the kinase compound protein interaction dataset in order to directly compare to Hie et al. [14]. For the benefit of future studies, we include the regression versions of each dataset we chose not to use on our GitHub dataset page.

**RC:** ***166-168: Why are the hyperparameters tuned based on a small dataset (thiolase) rather than performing hyperparameter tuning on the largest dataset (i.e., phosphatases)?***

AR: We select the thiolase dataset as it has an intermediate number of enzymes (73) with respect to the rest of the data. The halogenase dataset is limited to 42 enzymes, whereas the phosphatase dataset contains 218 enzymes. Our goal in hyperparameter optimizing on a held out dataset was to reduce noise, keep computational cost and complexity of experiments reasonable, and also reduce any risk of overfitting hyperparameters to the same datasets being shown in results. As such, we avoided optimizing hyeprparmeters on the larger datasets themselves.

Hyperparameters control only a small number of model settings, such as the regularization coefficient. To demonstrate that this would not significantly sway our results and conclusions, we conducted a hyperparameter optimization over the phosphatase dataset in the enzyme discovery direction and subsequently benchmarked models using these new hyperparameters. The newly identified hyperparameters (Tab. R7) are surprisingly similar to those exported from the thiolase data, and as such, we find model performance and relative ranking to be near identical between hyperparameter choices (Fig. R1).

**RC:** ***And what is the predictive performance on thiolase dataset after hyperparameter tuning?***

AR: To include this new information, we have updated the Supporting Information results tables to now feature an additional column for the hyperparameter optimization dataset (Tab. R5) and have updated the main text to reference these tables when describing hyperparameter optimization.

> We optimize model hyperparameters on the thiolase dataset[24] prior to training and evaluating on the remaining five datasets. We additionally report benchmarking performance for the thiolase dataset in the Supporting Information (Tab. S5, S6).

**RC:** ***174-178: Are there explanations on why CPI models do not outperform single-task models given that CPI models have been trained on both enzymes and substates as inputs and a greater amount of data? What is the AUPRC of CPI models using KRR as top model instead of FFN? Is this due to dissimilarities in the substrate structures or enzyme sequences maybe?***

AR: While there are many plausible explanations for why such an effect may observe, we hypothesize that joint CPI models may be predicting *averages* of new enzyme activity across all tasks. We refer the reviewer to our explanation in the following substrate discovery section:

> Curiously, in both the enzyme discovery (Fig. S9 S10, S8, S7, S11) and substrate discovery (Fig. S13, S12, S14) settings, predictions made by CPI models exhibit far more "blocky" characteristics than the respective single task models: when extrapolating to new enzymes, the prediction variance is not sensitive to the paired substrate for CPI models. This indicates that our CPI models struggle to condition their predictions to new enzymes (substrates) based upon the substrate (enzyme) pairing.

Regarding different KRR models, we primarily investigate binary classification tasks, and as such are not able to use continuous regression models. We believe that the various neural network architectures we utilize account for different "top" models and ways to combine enzyme sequence and substrate features.

**RC:** ***198-200: Similarly, what is the predictive performance on halogenase dataset after hyperparameter tuning?***

AR: As noted above with the thiolase dataset, we also add an additional column to our Supporting information results tables for the substrate discovery setting, reproduced in Table R6.

> In this case, we restrict our analysis only to the glycosyltransferase and phosphatase datasets where the number of substrates is $> 50$, using the halogenase dataset to tune hyperparameters for each model. We report the results comparison on the halogenase dataset in the Supporting Information (S7, S8).

**RC:** ***Figure 5B: It seems that those AUPRC's shown are for the sequence discovery tasks, according to baseline values in the AUPRC plots. For the last figure, the 0.42 Spearman's R baseline for kinase dataset seems to be that of the substrate discovery task. Please specify this in the caption.***

We have updated the caption accordingly to make clear that all plots shown are for enzyme discovery, except for the kinases, which represent drug repurposing. We also specify that we show Spearman's $\rho$ because the kinase dataset contains continuous, not binarized, values unlike the rest of our dataset.

> B) Enzyme discovery AUPRC are computed for various different pooling strategies. Each strategy is tested for different thresholds of residues to pool, comparing against both KNN Levenshtein distance baselines and a mean pooling baseline. The same hyperparameters are used as set in Figure 2 for ridge regression models. The kinase repurposing regression task from Hie et al. is shown with Spearman's $\rho$ instead of AUPRC as interactions are continuous, not binarized.. . .

**RC:** ***Also, kinase's sequence discovery task results are also interesting and should be discussed.***

AR: We have added an additional sentence to our results section discussing the kinase drug repurposing task and results, including a potential explanation for the increase in active site pooling performance:

> In the case of the kinases, the Levenshtein distance baseline outperforms the mean pooling method with respect to Spearman rank correlation, but using active site aware pooling outperforms both. We find that the performance increase is steeper in the kinase repurposing setting compared to other datasets, potentially due to both the regression nature of the dataset and also the non-dynamic binding of compound inhibitors in comparison to the other tasks, which focus on enzymatic catalysis not protein inhibition.. . .

**RC:** ***58-60: Consider citing this reference for enzyme identification: https://link.springer.com/article/10.1186/1752-0509-4-35***

AR: We have added this additional reference earlier in the text to augment our discussion of previous enzyme selection strategies:

> . . . Current enzyme selection methods often use simple similarity searches, comparing the desired reaction to precedent reactions in a database[6] [7].

**RC:** ***127-128: The argument that the novel substrates and sequence prediction is tough is well taken but nevertheless the authors could add some data to show this.***

AR: We have added a reference to Hie et al.'s previous compound protein interaction work. They consider the task of novel compound and novel protein extrapolation and plot performance explicitly in their supplement, in which they observe worse performance than novel compound or novel protein prediction:

> We omit the more difficult problem of generalizing to new substrates and new enzymes simultaneously; we posit that we must first be able to generalize in each direction separately in order to generalize jointly and note that empirical performance on joint generalization in compound protein interaction is lower in previous CPI studies [14].

**RC:** ***189-190: Can the authors please refer to this plot 2E here ? May be rewrite "Plotting the AUPRC metric as a function of number of actives"***

AR:

> Plotting the AUPRC metric as a function of number of active enzyme-substrate pairs reveals a strong positive correlation, validating that the number of hits observed in the training set will largely determine the success of the model in generalizing beyond the training set (Fig. 2E).

**RC:** ***259-260: Authors point to this important issue in the field. There has been some recent work that has indicated that random emeddings can also lead to useful predictions sometimes mitigating the value of language models and perhaps authors can cite this work here.***

AR: We thank the reviewer for calling attention to these other observations in the field. We have added two additional references suggested by Reviewer 2 to address these concerns:

1. Mean pooling may be sub-optimal:

   > However, for locally-defined properties, such as enzymatic catalysis or ligand binding at an active site, this mean pooling strategy may be sub-optimal [10].

2. Simple baselines are necessary in comparison to language models:

   > Our findings are retrospective in nature and do not negate the value of prospective experimental validation[14], but rather make clear that the field requires new methods to leverage the rich information contained in protein, small molecule interaction screens and truly learn interactions. This further reinforces the necessity for simple baseline models in protein engineering studies [25, 19].

**RC:** ***269-270: Could you provide more details about how the different "reference structures" you provided are combined for identifying active site in your proposed methods of structure-based active site pooling strategy?***

AR: We have added an additional reference to the more complete description of active site pooling in our Methods section, where we describe this in more detail.

> We rank order the columns in the MSA to be pooled based upon the (i) proximity to the active site of a single "reference structure" (Section "Active site pooling")...

**RC:** ***The Github page does not have the details of some parts of the code needed to reproduce the results though it has the datasets information.***

AR: As described above, we refer the reviewer to our second GitHub repository released with our manuscript: `https://github.com/samgoldman97/enz-pred`. We intentionally separate our repositories as

we believe the datasets and models represent independent contributions, and other scientists may be interested in *either* our models or the datasets. By separating the datasets into their own repository, we hope to more prominently feature each contribution and increase access to the data.

We highlight the location in the main text where we reference this second repository within *Methods* subsection *Code and Data Availability*:

> All code and enzyme data can be found on GitHub at `https://github.com/samgoldman97/enzyme-datasets`. Code to reproduce models can be found at `https://github.com/samgoldman97/enz-pred`. Code to reproduce the reanlaysis on the kinase data from Davis et al. can be found at `https://github.com/samgoldman97/kinase-cpi-reanalysis`.

## References


[1] Ethan C Alley, Grigory Khimulya, Surojit Biswas, Mohammed AlQuraishi, and George M Church. Unified rational protein engineering with sequence-based deep representation learning. *Nature methods*, 16(12):1315–1322, 2019. Publisher: Nature Publishing Group.

[2] Tristan Bepler and Bonnie Berger. Learning protein sequence embeddings using information from structure. *arXiv preprint arXiv:1902.08661*, 2019.

[3] Tristan Bepler and Bonnie Berger. Learning the protein language: Evolution, structure, and function. *Cell Systems*, 12(6):654–669, 2021.

[4] Surojit Biswas, Grigory Khimulya, Ethan C. Alley, Kevin M. Esvelt, and George M. Church. Low-N protein engineering with data-efficient deep learning. *bioRxiv*, 2020. Publisher: Cold Spring Harbor Laboratory _eprint: https://www.biorxiv.org/content/early/2020/01/24/2020.01.23.917682.full.pdf.

[5] Drew H Bryant, Ali Bashir, Sam Sinai, Nina K Jain, Pierce J Ogden, Patrick F Riley, George M Church, Lucy J Colwell, and Eric D Kelsic. Deep diversification of an aav capsid protein by machine learning. *Nature Biotechnology*, 39(6):691–696, 2021.

[6] Pablo Carbonell, Jerry Wong, Neil Swainston, Eriko Takano, Nicholas J Turner, Nigel S Scrutton, Douglas B Kell, Rainer Breitling, and Jean-Loup Faulon. Selenzyme: Enzyme selection tool for pathway design. *Bioinformatics*, 34(12):2153–2154, 2018.

[7] Ayoun Cho, Hongseok Yun, Jin Hwan Park, Sang Yup Lee, and Sunwon Park. Prediction of novel synthetic pathways for the production of desired chemicals. *BMC Systems Biology*, 4(1):1–16, 2010.

[8] Christian Dallago, Jody Mou, Kadina E Johnston, Bruce J Wittmann, Nicholas Bhattacharya, Samuel Goldman, Ali Madani, and Kevin K Yang. Flip: Benchmark tasks in fitness landscape inference for proteins. 2021.

[9] Christian Dallago, Konstantin Schütze, Michael Heinzinger, Tobias Olenyi, Maria Littmann, Amy X Lu, Kevin K Yang, Seonwoo Min, Sungroh Yoon, James T Morton, et al. Learned embeddings from deep learning to visualize and predict protein sets. *Current Protocols*, 1(5):e113, 2021.

[10] Nicki Skafte Detlefsen, Søren Hauberg, and Wouter Boomsma. What is a meaningful representation of protein sequences? *arXiv preprint arXiv:2012.02679*, 2020.

[11] Jacob Devlin, Ming-Wei Chang, Kenton Lee, and Kristina Toutanova. Bert: Pre-training of deep bidirectional transformers for language understanding. *arXiv preprint arXiv:1810.04805*, 2018.

[12] Vanessa E Gray, Ronald J Hause, Jens Luebeck, Jay Shendure, and Douglas M Fowler. Quantitative missense variant effect prediction using large-scale mutagenesis data. *Cell systems*, 6(1):116–124, 2018.

[13] Michael Heinzinger, Ahmed Elnaggar, Yu Wang, Christian Dallago, Dmitrii Nechaev, Florian Matthes, and Burkhard Rost. Modeling aspects of the language of life through transfer-learning protein sequences. *BMC bioinformatics*, 20(1):1–17, 2019.

[14] Brian Hie, Bryan D. Bryson, and Bonnie Berger. Leveraging uncertainty in machine learning accelerates biological discovery and design. *Cell Systems*, 11(5):461–477. e9, 2020. ISBN: 2405-4712 Publisher: Elsevier.

[15] Kexin Huang, Tianfan Fu, Wenhao Gao, Yue Zhao, Yusuf Roohani, Jure Leskovec, Connor W Coley, Cao Xiao, Jimeng Sun, and Marinka Zitnik. Therapeutics data commons: machine learning datasets and tasks for therapeutics. *arXiv preprint arXiv:2102.09548*, 2021.

[16] Wengong Jin, Regina Barzilay, and Tommi Jaakkola. Junction tree variational autoencoder for molecular graph generation. In *International Conference on Machine Learning*, pages 2323–2332. PMLR, 2018.

[17] Paul Kim, Robin Winter, and Djork-Arné Clevert. Deep protein-ligand binding prediction using unsupervised learned representations. *ChemRxiv*, 2020.

[18] Shuya Li, Fangping Wan, Hantao Shu, Tao Jiang, Dan Zhao, and Jianyang Zeng. MONN: a multi-objective neural network for predicting compound-protein interactions and affinities. *Cell Systems*, 10(4):308–322. e11, 2020. ISBN: 2405-4712 Publisher: Elsevier.

[19] Tianyu Lu, Alex X Lu, and Alan M Moses. Random embeddings and linear regression can predict protein function. *arXiv preprint arXiv:2104.14661*, 2021.

[20] Harry L. Morgan. The generation of a unique machine description for chemical structures-a technique developed at chemical abstracts service. *Journal of Chemical Documentation*, 5(2):107–113, 1965. ISBN: 0021-9576 Publisher: ACS Publications.

[21] Roshan Rao, Nicholas Bhattacharya, Neil Thomas, Yan Duan, Peter Chen, John Canny, Pieter Abbeel, and Yun Song. Evaluating protein transfer learning with TAPE. In *Advances in Neural Information Processing Systems*, pages 9689–9701, 2019.

[22] Roshan M Rao, Joshua Meier, Tom Sercu, Sergey Ovchinnikov, and Alexander Rives. Transformer protein language models are unsupervised structure learners. *bioRxiv*, 2020. Publisher: Cold Spring Harbor Laboratory _eprint: https://www.biorxiv.org/content/early/2020/12/15/2020.12.15.422761.full.pdf.

[23] Alexander Rives, Joshua Meier, Tom Sercu, Siddharth Goyal, Zeming Lin, Jason Liu, Demi Guo, Myle Ott, C. Lawrence Zitnick, Jerry Ma, and Rob Fergus. Biological structure and function emerge from scaling unsupervised learning to 250 million protein sequences. *Proceedings of the National Academy of Sciences*, 118(15):e2016239118, April 2021.

[24] Serina L. Robinson, Megan D. Smith, Jack E. Richman, Kelly G. Aukema, and Lawrence P. Wackett. Machine learning-based prediction of activity and substrate specificity for OleA enzymes in the thiolase superfamily. *Synthetic Biology*, 5(1), January 2020. Publisher: Oxford Academic.

[25] Amir Shanehsazzadeh, David Belanger, and David Dohan. Is transfer learning necessary for protein landscape prediction? *arXiv preprint arXiv:2011.03443*, 2020.
